# A real-world comparison of transcatheter edge-to-edge repair versus surgical mitral valve intervention in patients with mitral regurgitation: a TriNetX study

**DOI:** 10.3389/fmed.2026.1729871

**Published:** 2026-02-09

**Authors:** Kuan-Chieh Tu, Jheng-Yan Wu

**Affiliations:** 1 Division of Cardiology, Department of Internal Medicine, Chi Mei Medical Center, Tainan, Taiwan; 2 Department of Nutrition, Chi Mei Medical Centre, Tainan, Taiwan; 3 Department of Public Health, College of Medicine, National Cheng Kung University, Tainan, Taiwan

**Keywords:** all-cause mortality, mitral regurgitation, mitral transcatheter edge-to-edge repair, mitral valve surgery, transcatheter edge-to-edge repair

## Abstract

**Objective:**

We aimed to compare the clinical outcomes of transcatheter edge-to-edge repair (TEER) and surgical repair in patients with non-rheumatic mitral regurgitation (MR) using real-world data.

**Methods:**

We conducted a retrospective cohort study using TriNetX to identify adults with mitral regurgitation who underwent surgical repair or TEER (2013–2025). Propensity score matching (PSM) balanced demographics, comorbidities, and medications. The primary outcome was all-cause mortality; the secondary outcomes included cardiovascular events.

**Results:**

After exclusions, 35,753 patients with mitral regurgitation were identified, including 2,165 TEER and 33,588 surgical cases; 2,029 matched pairs were analyzed. At 1 year, surgery showed lower mortality (hazard ratio [HR]: 0.78; 95% confidence interval [CI]: 0.64–0.96), primarily between 2 months and 1 year. Surgery was associated with higher rates of early dyspnea, atrial fibrillation, and heart failure, though heart failure could be reversed later. No significant differences were found in major adverse cardiovascular events (MACEs), stroke, acute myocardial infarction (AMI), cardiac arrest, or emergency department (ED) visits. A subgroup analysis of patients with mitral valve prolapse showed consistent results, with atrial fibrillation and heart failure occurring more frequently after surgery.

**Conclusion:**

In this large cohort, surgery and TEER produced different outcomes. Early mortality was similar, but surgery improved survival after 2 months. TEER had fewer early dyspnea, atrial fibrillation, and heart failure events. No differences were observed in MACE, stroke, AMI, or ED visits, supporting individualized MR treatment.

## Introduction

Mitral regurgitation (MR) is a valvular heart disease characterized by inadequate coaptation of the mitral valve leaflets, resulting in systolic retrograde blood flow from the left ventricle into the left atrium. It affects approximately 2% of the global population (1–3). The pathophysiological process can lead to pulmonary edema and remodeling of the left ventricle and pulmonary vasculature (4). Numerous studies have demonstrated that MR is associated with increased rates of hospitalization and mortality (5).

The etiology of MR can be broadly classified as primary (e.g., leaflet prolapse or flail) or secondary (functional MR) causes (6). For patients with severe primary MR, surgical intervention—such as mitral valve repair, annuloplasty, or valve replacement—has traditionally been the mainstay of treatment (7). However, some patients with significant comorbidities or high surgical risk are not suitable candidates for open-heart surgery. In recent years, transcatheter edge-to-edge repair (TEER) has emerged as a less invasive alternative for these high-risk patients (8). TEER involves the percutaneous delivery of a mechanical grasping device into the left atrium via a transseptal approach to approximate the mitral valve leaflets, thereby creating a double-orifice valve. This technique is based on the Alfieri stitch concept and reduces MR by improving leaflet coaptation directly and exerting an indirect annuloplasty-like effect (9, 10). The Endovascular Valve Edge-to-Edge Repair Study II (EVEREST II) trial compared percutaneous mitral valve repair with surgical repair in patients with moderate-to-severe primary MR, demonstrating that percutaneous repair was less effective than surgical repair, primarily due to a higher rate of repeat mitral valve surgeries in the percutaneous group (11, 12). Observational studies have shown that TEER is associated with reduced rates of rehospitalization, improved functional status, a significant reduction in MR severity, and favorable ventricular remodeling in patients deemed to be at prohibitive surgical risk (13, 14). Accordingly, the most recent European Society of Cardiology (ESC) guidelines continued to recommend TEER as Class IIa for patients with severe primary MR who are at high surgical risk (15). In contrast, the effectiveness of mitral TEER for secondary MR was first established in the Cardiovascular Outcomes Assessment of the MitraClip Percutaneous Therapy (COAPT) trial, which demonstrated superiority over optimal medical therapy, and was further supported by the RESHAPE-HF trial (16, 17). More recently, the randomized MATTERHORN trial directly compared mitral TEER with surgical mitral repair and demonstrated that TEER is non-inferior to surgical mitral repair in patients with heart failure and secondary mitral regurgitation (18). Reflecting this expanding evidence base, the updated ESC guidelines have upgraded TEER to a Class I recommendation for patients with ventricular secondary MR who meet COAPT-like criteria, while assigning a Class IIb recommendation for those who do not fully meet COAPT-like criteria or who have atrial functional MR (15).

However, real-world data on the efficacy and safety of TEER in patients with non-rheumatic MR remain limited. To address this gap, we conducted a retrospective cohort study using data from the TriNetX Research Network, which includes 48 healthcare organizations (HCOs) across the United States, to compare the clinical outcomes of TEER and surgical repair in patients with non-rheumatic MR.

## Methods

### Data source

This retrospective cohort study used data from TriNetX, a global federated health research network comprising electronic health records from approximately 175 million patients across 153 HCOs. The database provides comprehensive clinical information, such as diagnostic and procedural codes, medication prescriptions, laboratory test results, and genomic data. TriNetX enables secure, real-time access to de-identified, aggregated data from a demographically and geographically diverse patient population spanning hospitals, primary care settings, and specialty care institutions. The platform operates under a waiver granted by the Western Institutional Review Board, as it provides only aggregate-level statistical outputs without access to individual-level identifiers. The present study was conducted in accordance with the Strengthening the Reporting of Observational Studies in Epidemiology (STROBE) guidelines.

### Study design

We included adult patients (aged ≥18 years) with a documented diagnosis of non-rheumatic mitral valve prolapse and insufficiency, identified using the International Classification of Diseases, Tenth Revision, Clinical Modification (ICD-10-CM) codes I34.0 and I34.1, who initiated treatment with either surgical repair or TEER between 1 January 2013 and 30 June 2025. The index date was defined as the date of the first recorded intervention corresponding to the assigned treatment group. Patients with exposure to both TEER and surgery at any point were excluded to ensure a comparison between single-modality treatment pathways. Individuals undergoing TEER followed by subsequent surgery or vice versa were therefore not retained in the analytical cohort. Procedural granularity, including the number of TEER clips, device generation, surgical repair versus replacement, and residual MR severity, was not available within TriNetX and, therefore, could not be incorporated into exposure or subgroup definitions. Patients with any prior or concurrent exposure to the alternative treatment modality, either before or after the index date, were excluded. Additionally, individuals with recorded occurrences of the study outcomes prior to the index date were excluded. Detailed definitions and coding algorithms for all study variables are provided in Supplementary Table S1.

### Covariates and propensity score matching (PSM)

Based on the defined cohort criteria, index dates, outcome measures, and potential confounders, a covariate matrix was constructed using patient-level data from the 12-month period preceding the index date. Propensity scores were then estimated via a logistic regression analysis to model the probability of receiving the comparator treatment conditional on baseline covariates. A one-to-one nearest-neighbor matching algorithm was implemented using a greedy matching approach, with a caliper width set at 0.1 times the pooled standard deviation (SD) of the logit-transformed propensity score. Patients in the smaller treatment group were matched to the most similar individuals in the larger group. Covariate balance between the matched cohorts was evaluated using standardized mean differences (SMDs), with an SMD of <0.1 considered indicative of adequate balance (19). Matching was performed without replacement, and all outcome analyses were conducted within the matched cohorts after propensity score matching (PSM) to avoid imbalance introduced by the unmatched population.

The propensity score matching incorporated a comprehensive set of baseline variables to ensure comparability between treatment groups. Demographic variables included age at index (mean and standard deviation), sex (female or male), and ethnicity categorized as white, Black or African American, Asian, other, or unknown. Clinical comorbidities included type 2 diabetes, hypertension, dyslipidemia, overweight and obesity, chronic kidney disease, nicotine dependence, alcohol-related disorders, chronic lower respiratory diseases, ischemic heart diseases, cerebrovascular diseases, and neoplasms. Baseline medication use was also included as a covariate, specifically angiotensin-converting enzyme inhibitors (ACEi), angiotensin II receptor blockers (ARBs), diuretics, digitalis glycosides, eplerenone, ivabradine, ezetimibe, vericiguat, evolocumab, and alirocumab. Glycemic control was assessed using hemoglobin A1c, reported as a continuous variable (mean and standard deviation), with an additional binary indicator for glycated hemoglobin (HbA1c) ≥ 9%. Detailed definitions and coding algorithms for all covariates are provided in Supplementary Table S2.

### Outcomes and follow-up

The primary outcome was all-cause mortality. Secondary outcomes included all-cause emergency department (ED) visits, dyspnea, mitral stenosis, atrial fibrillation, major adverse cardiovascular events (MACEs), stroke, acute myocardial infarction (AMI), cardiac arrest, and worsening heart failure. Follow-up commenced the day after the index date and continued until the earliest occurrence of any of the following: the occurrence of any outcome event, the date of the last recorded clinical encounter, death, or 1 year post-index, whichever occurred first. Comprehensive definitions and coding algorithms for all outcomes are detailed in Supplementary Table S3.

### Statistical analysis

Continuous variables were reported as means with standard deviations, and categorical variables were presented as frequencies with corresponding percentages. To mitigate potential confounding and enhance comparability between treatment groups, PSM was performed before the primary and subgroup analyses. Time-to-event outcomes were evaluated using Cox proportional hazards models to estimate hazard ratios (HRs) with 95% confidence intervals (CIs). Differences in event-free survival between groups were assessed using the Kaplan–Meier survival analysis and compared via log-rank tests.

To evaluate the robustness of the observed associations, E-values were calculated to quantify the minimum strength of association that an unmeasured confounder would need to have with both the exposure and the outcome to completely explain the observed effect estimates (20). Beyond the primary analysis covering the 1-day to 1-year follow-up period, we also conducted interval-specific analyses for 1 day to 2 months and 2 months to 1 year. In addition, a landmark-style temporal evaluation was performed by separating outcomes into early (1-day to 1-month) and late (1-month to 1-year) follow-up windows to distinguish peri-procedural effects from later clinical divergence. A sensitivity analysis limited to procedures performed between 2018 and 2025 was additionally conducted to evaluate temporal, learning-curve, and device-generation effects. All statistical analyses were performed using the TriNetX analytics platform.

## Results

**Figure 1:**
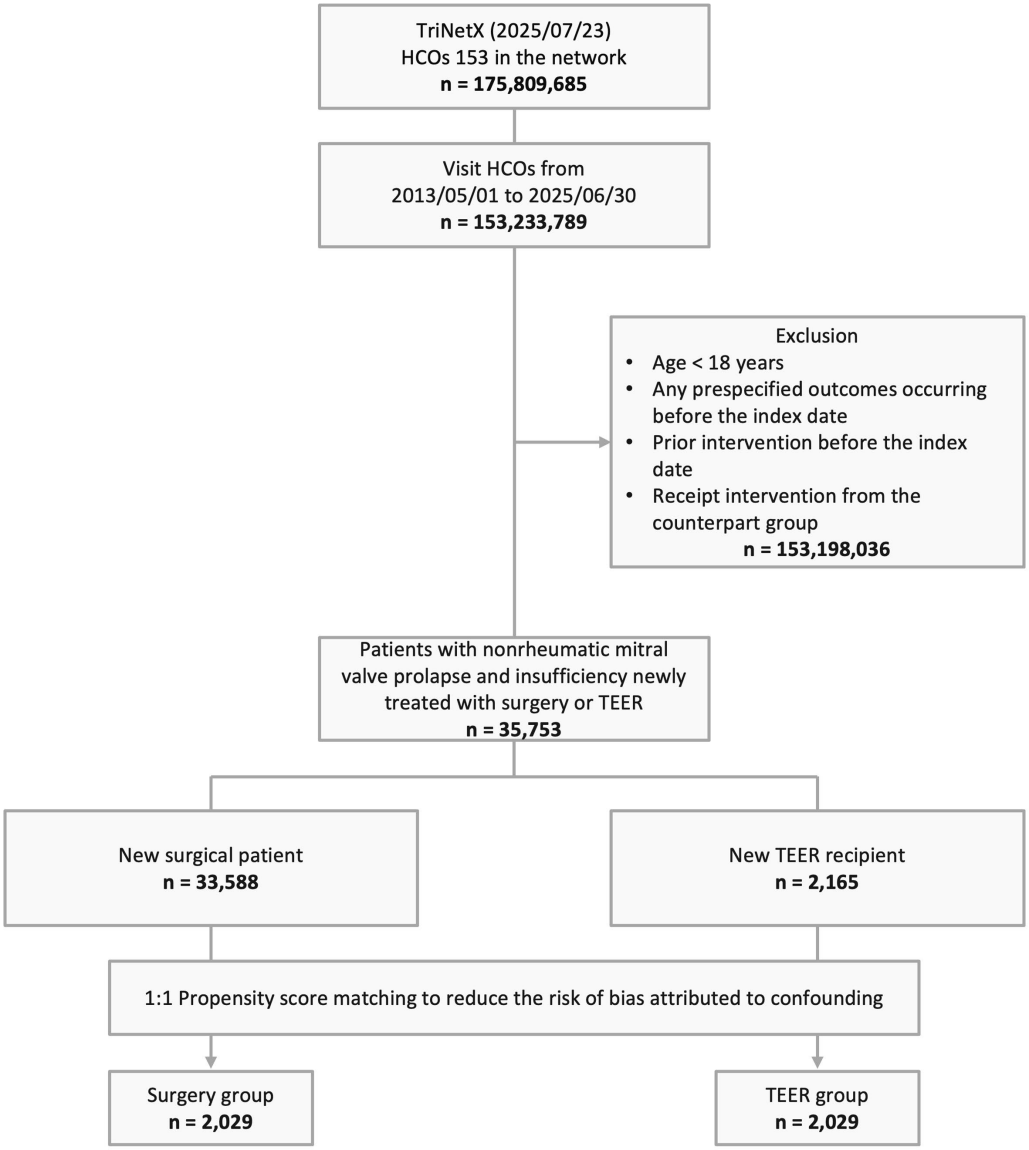
Study design and selection flow. HCOs, healthcare organizations; TEER, transcatheter edge-to-edge mitral valve repair.

After excluding patients younger than 65 years, those with a prespecified outcome before the index date, and those with a history of prior mitral valve intervention, a total of 35,753 patients with mitral valve insufficiency were included in this study. Of these patients, 33,588 underwent surgical intervention, while 2,165 underwent TEER during the study period (Figure 1).

Before matching, the surgical group exhibited a higher prevalence of dyslipidemia, overweight/obesity, nicotine dependence, alcohol-related disorders, ischemic heart disease, and cerebrovascular disease. In contrast, patients in the TEER group more frequently used diuretics and digitalis. After PSM, 2,029 patients were allocated to each group, with no significant differences in baseline characteristics. These matched cohorts were subsequently included in the statistical analyses. Detailed baseline characteristics are presented in Table 1.

**Table 1:** Baseline characteristics of the included subjects.

Variables	Before matching			After matching		
	Surgery group (*n* = 33,588)	TEER group (*n* = 2,165)	Standardized difference	Surgery group (*n* = 2,029)	TEER group (*n* = 2,029)	Standardized difference
Age at index, years						
Mean (SD)	62.6 (13.8)	76.6 (10.5)	1.135	75.4 (9)	75.9 (10.5)	0.048
Sex, *n* (%)						
Female	13,979 (42.1)	950 (43.9)	0.035	867 (42.7)	899 (44.3)	0.032
Male	17,691 (53.3)	1,176 (54.3)	0.02	1,120 (55.2)	1,091 (53.8)	0.029
Ethnicity, *n* (%)						
White	24,523 (73.9)	1,542 (71.2)	0.06	1,443 (71.1)	1,477 (72.8)	0.037
Black or African American	3,285 (9.9)	231 (10.7)	0.025	208 (10.3)	212 (10.4)	0.006
Asian	1,015 (3.1)	32 (1.5)	0.106	21 (1)	30 (1.5)	0.04
Other ethnicity	706 (2.1)	36 (1.7)	0.034	43 (2.1)	33 (1.6)	0.036
Unknown ethnicity	3,352 (10.1)	321 (14.8)	0.143	310 (15.3)	274 (13.5)	0.051
Comorbidities, *n* (%)						
Type 2 diabetes	6,391 (19.3)	360 (16.6)	0.069	321 (15.8)	342 (16.9)	0.028
Hypertension	17,197 (51.8)	1,032 (47.7)	0.083	979 (48.3)	989 (48.7)	0.01
Dyslipidemia	16,176 (48.7)	878 (40.6)	0.165	803 (39.6)	855 (42.1)	0.052
Overweight and obesity	5,521 (16.6)	205 (9.5)	0.214	217 (10.7)	203 (10)	0.023
Chronic kidney disease	5,882 (17.7)	576 (26.6)	0.215	546 (26.9)	527 (26)	0.021
Nicotine dependence	4,110 (12.4)	89 (4.1)	0.304	98 (4.8)	88 (4.3)	0.024
Alcohol-related disorders	1,150 (3.5)	29 (1.3)	0.139	30 (1.5)	29 (1.4)	0.004
Chronic lower respiratory diseases	6,674 (20.1)	390 (18)	0.053	358 (17.6)	372 (18.3)	0.018
Ischemic heart diseases	18,182 (54.8)	1,038 (47.9)	0.137	992 (48.9)	999 (49.2)	0.007
Cerebrovascular diseases	6,718 (20.2)	214 (9.9)	0.293	198 (9.8)	213 (10.5)	0.025
Neoplasms	4,997 (15.1)	352 (16.3)	0.033	332 (16.4)	331 (16.3)	0.001
Medications, *n* (%)						
ACEi	7,540 (22.7)	454 (21)	0.042	408 (20.1)	437 (21.5)	0.035
ARBs	5,745 (17.3)	580 (26.8)	0.23	560 (27.6)	526 (25.9)	0.038
Diuretic	17,822 (53.7)	1,423 (65.7)	0.247	1,306 (64.4)	1,313 (64.7)	0.007
Digitalis glycosides	2,165 (6.5)	226 (10.4)	0.141	221 (10.9)	196 (9.7)	0.041
Eplerenone	116 (0.4)	25 (1.2)	0.093	25 (1.2)	22 (1.1)	0.014
Ivabradine	67 (0.2)	14 (0.6)	0.068	18 (0.9)	10 (0.5)	0.048
Ezetimibe	723 (2.2)	101 (4.7)	0.137	78 (3.8)	90 (4.4)	0.03
Vericiguat	10 (0)	10 (0.5)	0.087	10 (0.5)	10 (0.5)	0
Evolocumab	73 (0.2)	10 (0.5)	0.042	10 (0.5)	10 (0.5)	0
Alirocumab	24 (0.1)	10 (0.5)	0.076	10 (0.5)	10 (0.5)	0
Hemoglobin A1c, %						
Mean (SD)	5.8 (1.2)	6.1 (1.1)	0.236	5.8 (1.2)	6.1 (1.1)	0.217
≥9	825 (2.5)	40 (1.8)	0.044	41 (2)	39 (1.9)	0.007

ACEi, angiotensin-converting enzyme inhibitor; ARB, angiotensin receptor blocker; standard deviation, SD; TEER, transcatheter edge-to-edge mitral valve repair. A standardized difference < 0.1 is considered a small difference.

### Primary outcome

**Figure 2:**
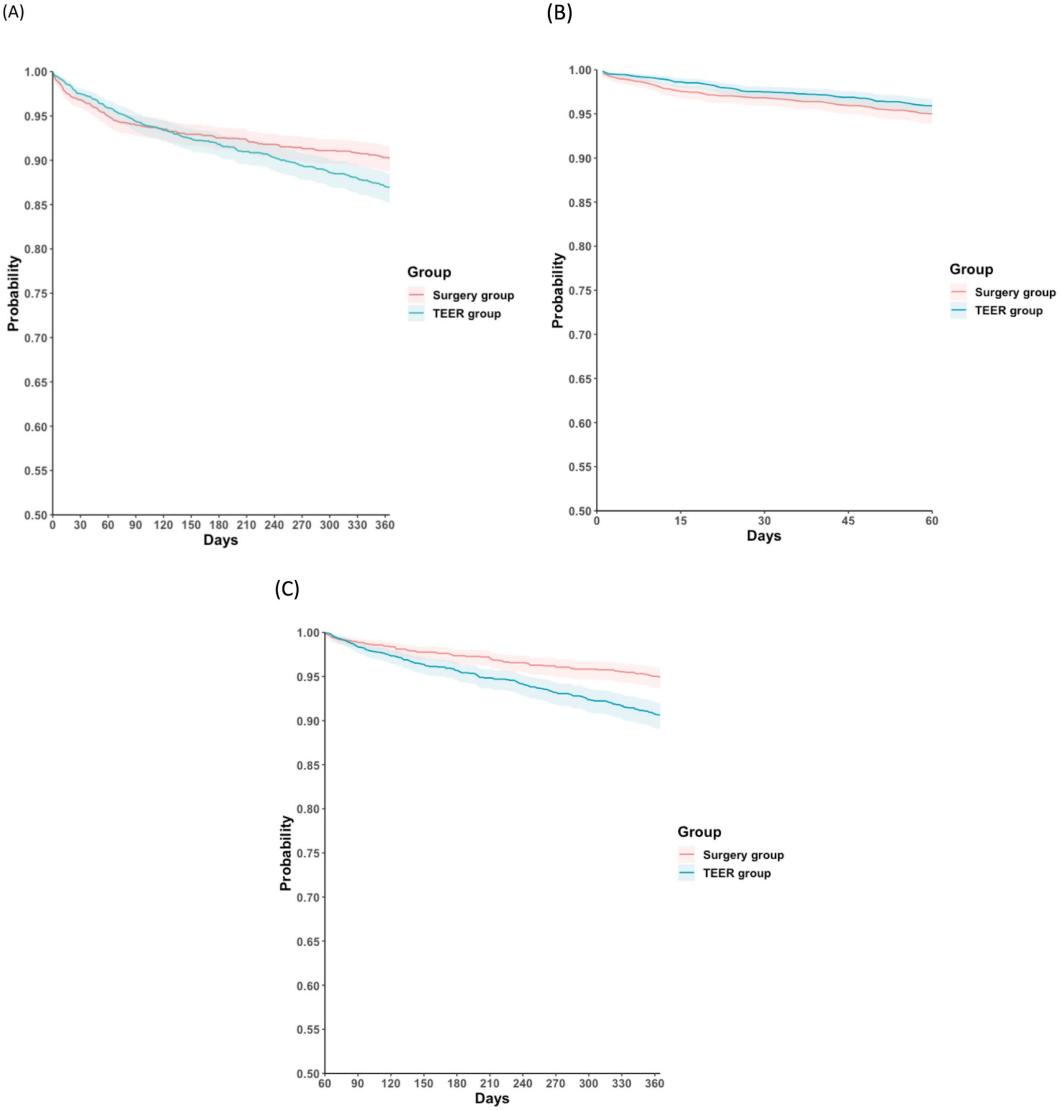
Kaplan–Meier time-to-event free curves for all-cause mortality in the surgery and the TEER groups at different follow-up intervals: **(A)** 1 day and 1 year; **(B)** 1 day and 2 months; **(C)** 2 months and 1 year. TEER, transcatheter edge-to-edge mitral valve repair.

Tables 2–4 summarize the clinical outcomes between the two groups at different post-procedural time intervals. During follow-up, 166 patients in the surgery group (8.2 events per 100 person-years [PYs]) and 220 patients in the TEER group (10.9 events per 100 PYs) died. The surgery group had a lower 1-year mortality rate than the TEER group (HR: 0.78; 95% CI: 0.64–0.96; *p* = 0.017; Figure 2A). Temporal analysis suggested that this difference in survival occurred primarily during the intermediate postoperative phase, specifically between 2 months and 1 year after the index date (HR: 0.54; 95% CI: 0.41–0.71; *p* < 0.001; Figure 2B). In contrast, no significant difference in mortality was observed during the early postoperative period (day 1 to 2 months) (HR: 1.38; 95% CI: 0.90–2.11; *p* = 0.134; Figure 2C). Interpretation should consider that clip number, device iteration, and surgical technique (repair versus replacement) could not be stratified in this dataset. Residual MR after TEER was also unavailable, which may influence early symptoms and HF patterns. To further clarify peri-procedural versus later mortality risk, early and late intervals were summarized using a 1-month landmark structure. Supplementary Table S4 presents event counts and hazard ratios during the early postoperative phase (day 1–1 month), while Supplementary Table S5 reports outcomes from 1 month to 1 year. These stratified data reinforce the temporal divergence observed in the main analysis, showing a minimal difference in early mortality but a pronounced advantage favoring surgery during later follow-up. In the post-2018 restricted cohort, which represents mature TEER practice, mortality differences remained consistent (HR: 0.58; 95% CI: 0.45–0.73) (Supplementary Table S6).

**Table 2:** Hazard ratios of outcomes between the surgery and the TEER groups at the 1-day and 1-year follow-ups after the index date.

Outcome	Surgery group (*n* = 2,029)		TEER group (*n* = 2,029)		HR (95% CI)	*p*-value	E-value (95% LCL)
	Events (*n*)	Incidence rate per 100 PYs	Events (*n*)	Incidence rate per 100 PYs			
Primary outcome							
All-cause mortality	166	8.2	220	10.9	0.78 (0.64, 0.96)	0.017	1.9 (1.3)
Secondary outcomes							
All-cause ED visit	115	5.7	87	4.3	1.20 (0.91, 1.58)	0.203	1.7 (1.0)
Dyspnea	172	8.5	113	5.6	1.53 (1.21, 1.94)	<0.001	2.4 (1.7)
Mitral stenosis	18	0.9	55	2.7	0.35 (0.20, 0.59)	<0.001	5.2 (2.8)
Atrial fibrillation	156	7.7	150	7.4	1.91 (1.52, 2.38)	<0.001	3.2 (2.4)
MACEs	113	5.6	106	5.2	1.05 (0.81, 1.34)	0.723	1.3 (1.0)
Stroke	57	2.8	42	2.1	1.37 (0.92, 2.04)	0.123	2.1 (1.0)
AMI	74	3.6	68	3.4	1.06 (0.76, 1.48)	0.720	1.3 (1.7)
Cardiac arrest	23	1.1	21	1.0	1.12 (0.62, 2.02)	0.708	1.5 (1.0)
Worsening heart failure	645	31.8	151	7.4	4.88 (4.09, 5.83)	<0.001	9.2 (7.7)

AMI, acute myocardial infarction; CI, confidence interval; HR, hazard ratio; LCL, lower confidence limit; MACEs, major adverse cardiovascular events; PYs, person-years; TEER, transcatheter edge-to-edge mitral valve repair.

**Table 3:** Hazard ratios of outcomes between the surgery and the TEER groups at the 1-day and 2-month follow-ups after the index date.

Outcome	Surgery group (*n* = 2,029)		TEER group (*n* = 2,029)		HR (95% CI)	*p* value	E-value (95% LCL)
	Events (*n*)	Incidence rate per 100 PYs	Events (*n*)	Incidence rate per 100 PYs			
Primary outcome							
All-cause mortality	94	28.2	77	23.1	1.25 (0.92, 1.69)	0.149	1.8 (1.0)
Secondary outcomes							
All-cause ED visit	55	16.5	35	10.5	1.38 (0.90, 2.11)	0.134	2.1 (1.0)
Dyspnea	133	39.9	51	15.3	2.52 (1.82, 3.48)	<0.001	4.5 (3.1)
Mitral stenosis	14	4.2	37	11.1	0.40 (0.22, 0.74)	0.003	4.4 (2.0)
Atrial fibrillation	133	39.9	96	28.8	2.46 (1.89, 3.20)	<0.001	4.4 (3.2)
MACEs	69	20.7	52	15.6	1.28 (0.90, 1.84)	0.173	1.9 (1.0)
Stroke	33	9.9	19	5.7	1.72 (0.98, 3.02)	0.057	2.8 (1.0)
AMI	46	13.8	34	10.2	1.30 (0.83, 2.02)	0.246	1.9 (1.0)
Cardiac arrest	13	3.9	10	3.0	1.87 (0.75, 4.69)	0.175	3.2 (1.0)
Worsening heart failure	636	190.8	108	32.4	6.55 (5.34, 8.04)	<0.001	12.6 (10.2)

AMI, acute myocardial infarction; CI, confidence interval; HR, hazard ratio; LCL, lower confidence limit; MACEs, major adverse cardiovascular events; PYs, person-years; TEER, transcatheter edge-to-edge mitral valve repair.

**Table 4:** Hazard ratios of outcomes between the surgery and the TEER groups at the 2-month and 1-year follow-ups after the index date.

Outcome	Surgery group (*n* = 2,029)		TEER group (*n* = 2,029)		HR (95% CI)	*p* value	E-value (95% LCL)
	Events (*n*)	Incidence rate per 100 PYs	Events (*n*)	Incidence rate per 100 PYs			
Primary outcome							
All-cause mortality	73	3.6	143	7.1	0.54 (0.41, 0.71)	<0.001	1.9 (1.3)
Secondary outcomes							
All-cause ED visit	61	3.0	52	2.6	1.09 (0.75, 1.58)	0.648	1.4 (1.0)
Dyspnea	39	1.9	63	3.1	0.67 (0.45, 0.99)	0.046	2.4 (1.1)
Mitral stenosis	10	0.5	18	0.9	0.23 (0.08, 0.69)	0.004	8.2 (2.3)
Atrial fibrillation	24	1.2	54	2.7	0.89 (0.55, 1.44)	0.633	1.5 (1.0)
MACEs	44	2.2	54	2.7	0.82 (0.55, 1.22)	0.326	1.7 (1.0)
Stroke	24	1.2	23	1.1	1.07 (0.61, 1.90)	0.813	1.3 (1.0)
AMI	28	1.4	34	1.7	0.82 (0.50, 1.35)	0.439	1.7 (1.0)
Cardiac arrest	10	0.5	14	0.7	0.74 (0.33, 1.67)	0.465	2.0 (1.0)
Worsening heart failure	10	0.5	43	2.1	0.34 (0.17, 0.70)	0.002	5.3 (2.2)

AMI, acute myocardial infarction; CI, confidence interval; HR, hazard ratio; LCL, lower confidence limit; MACEs, major adverse cardiovascular events; PYs, person-years; TEER, transcatheter edge-to-edge mitral valve repair.

### Secondary outcomes

The surgery group was observed to have higher incidences of dyspnea (HR: 1.53; 95% CI: 1.21–1.94), atrial fibrillation (HR: 1.91; 95% CI: 1.52–2.38), and worsening heart failure (HR: 4.88; 95% CI: 4.09–5.83) compared with the TEER group. Dyspnea (HR: 2.52; 95% CI: 1.82–3.48; *p* < 0.001) and atrial fibrillation (HR: 2.46; 95% CI: 1.89–3.20; *p* < 0.001) were more frequent within the first 2 months post-procedure, but no significant differences were observed from 2 months to 1 year. Worsening heart failure was observed to be higher in the surgery group during the early postoperative period (HR: 6.55; 95% CI: 5.34–8.04; *p* < 0.001), but this pattern reversed between 2 months and 1 year, favoring the surgery group (HR: 0.34; 95% CI: 0.17–0.70; *p* = 0.002). E-value analysis indicated that unmeasured confounding was unlikely to be the sole explanation, with E-values (lower CI limits) of 1.9 (1.3) for all-cause mortality, 2.4 (1.7) for dyspnea, 5.2 (2.8) for mitral stenosis, and 3.2 (2.4) for atrial fibrillation.

No significant differences were observed between the two groups for all-cause ED visits (HR: 1.20; 95% CI: 0.91–1.50; *p* = 0.203), MACEs (HR: 1.05; 95% CI: 0.81–1.34; *p* = 0.723), stroke (HR: 1.37; 95% CI: 0.92–2.04; *p* = 0.123), AMI (HR: 1.06; 95% CI: 0.76–1.48; *p* = 0.720), or cardiac arrest (HR: 1.12; 95% CI: 0.62–2.02; *p* = 0.708).

### Subgroup analysis

**Figure 3:**
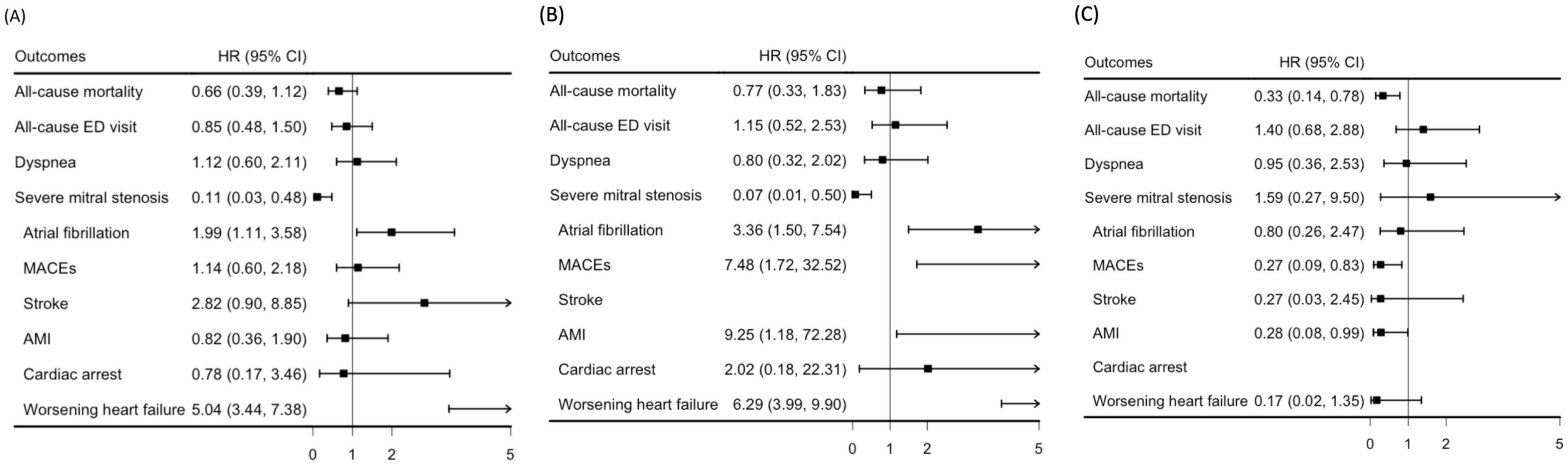
Subgroup analyses of the hazard ratios for the outcomes in the surgery and the TEER groups at different follow-up periods: **(A)** 1-day and 1-year, **(B)** 1-day and 2-months, and **(C)** 2-months and 1-year. AMI, acute myocardial infarction; CI, confidence interval; HR, hazard ratio; MACEs, major adverse cardiovascular events; TEER, transcatheter edge-to-edge mitral valve repair.

A subgroup analysis comparing the efficacy of TEER and surgical intervention in patients with mitral valve prolapse was performed. No statistically significant differences were observed between the groups for all-cause mortality, all-cause ED visits, dyspnea, MACEs, stroke, AMI, or cardiac arrest. However, atrial fibrillation and worsening heart failure were more frequent in the surgery group. Temporal analysis did not reveal any distinct trends between the two interventions (Figure 3).

## Discussion

In this retrospective observational study of a propensity-matched cohort of 4,118 patients with mitral valve regurgitation, TEER was associated with a higher risk of all-cause mortality compared with surgical mitral valve repair or replacement. In contrast, TEER was associated with significantly lower rates of dyspnea, atrial fibrillation, and worsening heart failure, with these benefits being most pronounced during the first 2 months post-procedure.

Surgical intervention remains the mainstay of treatment for mitral valve regurgitation. Reported 1-year mortality rates following mitral valve surgery vary widely, ranging from 9.1 to 27% (21–23), depending on patient comorbidities, the complexity of the surgical procedure, and the surgeon’s experience (24, 25). In patients with primary MR undergoing TEER, reported 1-year all-cause mortality varies across studies, ranging from approximately 5% in highly selected cohorts (using DragonFly devices) to up to 19.5% in high-risk, comorbid populations (26). Real-world registry data and meta-analyses have consistently shown a 1-year mortality rate of approximately 12–16% (21, 27). In the present observational study, the 1-year mortality rate of patients is 8.2 per 100 patient-years in the surgical group and 10.9 per 100 patient-years in the TEER group. A temporal analysis revealed that mitral valve surgery had similar mortality outcomes as mitral TEER intervention within the first 2 months after the index procedure, but demonstrated better survival thereafter. This observation is supported by the EVEREST II trial, and by multiple meta-analyses and propensity-matched studies, although the populations included in these studies were slightly different from the current cohort (11, 12, 28). In populations with comparable surgical risk, short-term (30-day or in-hospital) mortality rates are similar for mitral valve surgery and TEER, with both interventions exhibiting single-digit mortality rates. However, mitral valve surgery is associated with superior long-term outcomes.

In this retrospective cohort study, mitral valve surgical intervention was associated with higher incidences of atrial fibrillation, dyspnea, and worsening heart failure among patients with MR compared with mitral TEER. The temporal analysis revealed that these adverse outcomes predominantly occurred within the first 2 months following the index procedure. We believe that these early complications are largely related to perioperative events associated with open-heart mitral valve surgery. Large-scale, propensity-matched cohort data from France demonstrated similar results that TEER is associated with lower rates of procedural complications, such as atrial fibrillation and pulmonary edema, when compared to isolated mitral valve surgery (29). Randomized controlled evidence indicates that new-onset or persistent atrial fibrillation occurs more frequently following surgical mitral valve intervention compared with TEER, as demonstrated by the safety endpoints of the EVEREST II and MATTERHORN trials (18, 30). Regarding dyspnea and symptom burden, both treatment strategies yield substantial improvements. In EVEREST II, 12-month improvements in New York Heart Association functional class were comparable between groups, whereas MATTERHORN reported a greater 1-year improvement in Minnesota Living with Heart Failure Questionnaire scores in the TEER group (11, 18).

No significant differences were observed between groups for MACEs, stroke, or AMI in this comparative cohort study. Unlike the increased risk of cerebrovascular events observed with coronary artery bypass grafting versus percutaneous coronary intervention in patients with coronary artery disease, no such elevation was observed in the present cohort (31). However, during the first 2 months following the index procedure, a slightly higher rate of stroke was observed in the mitral surgical group, although this difference did not reach statistical significance. Across randomized and comparative studies, early cerebrovascular event rates after mitral valve interventions remain low and are generally comparable between strategies. In the EVEREST II trial, major stroke within 30 days occurred in 1% of patients undergoing TEER, compared with 2% of those who underwent surgery (12). Although a recent meta-analysis found no statistically significant difference in short-term stroke risk, the data suggested a numerical trend favoring TEER (21). Consistently, the MATTERHORN trial demonstrated the superior 30-day safety of TEER compared with surgery, based on a composite endpoint that included stroke (18).

### Limitations

This study has several important limitations. First, despite propensity matching, its retrospective observational design inherently exposes the study to potential selection bias, residual confounding, and unmeasured variables, such as New York Heart Association class, left ventricular ejection fraction (LVEF), left ventricular size, frailty, pulmonary pressures, and surgical risk (Society of Thoracic Surgeons score or European System for Cardiac Operative Risk Evaluation [EuroSCORE]). Second, the data were derived from the TriNetX database, which relies on aggregated electronic health records and International Classification of Diseases (ICD) coding; thus, the accuracy and completeness of diagnoses, procedures, and outcomes may be limited, potentially introducing misclassification or underreporting. Additionally, symptom-based outcomes such as dyspnea may be sensitive to coding variability. Third, as a retrospective observational study, the causal relationship between the interventions and the observed outcomes cannot be definitively established. The inherent limitations of observational data—such as residual confounding, unmeasured variables, and potential selection bias—make it challenging to fully clarify whether the differences between groups are directly attributable to the treatments themselves. Therefore, our findings should be interpreted with caution, and prospective randomized studies are needed to further validate these associations. Fourth, information regarding the specific type or manufacturer of Mitral TEER devices (e.g., clip type) or the exact surgical techniques for mitral valve repair or replacement was unavailable, preventing the analysis of device- or procedure-specific outcomes. Fifth, this study was unable to clearly distinguish the etiology of MR. Because patients who received both TEER and surgery were excluded, the analysis reflects outcomes among single-modality treatment courses only. TEER-to-surgery conversion events potentially representing clinical failure may therefore be underrepresented, which could bias the interpretation toward more favorable TEER-based symptom outcomes. Crossover-based sensitivity analyses were not feasible, as staged procedures could not be reliably distinguished from rescue conversions within TriNetX. Sixth, the calendar year of intervention was not available for inclusion in propensity matching, and temporal variation in TEER technology, operator experience, and surgical practice from 2013 to 2025 may have influenced the observed outcomes. Although a later-era restricted sensitivity analysis yielded similar risk patterns, residual learning-curve or device-generation effects cannot be fully excluded. Earlier TEER procedures may reflect different device generations and learning-curve phases compared with more recent practice, and results should therefore be interpreted in the context of potential temporal evolution. Additionally, numbers-at-risk beneath the Kaplan–Meier curves and spline-based knot visualization could not be generated because the TriNetX platform does not provide time-dependent censoring counts, covariate density overlays, or spline coefficients. Finally, granular procedural details, intraoperative findings, and imaging parameters were not captured, which could potentially influence both short- and long-term outcomes. While we attempted to mitigate confounding by using comedications and biochemical results as proxies for disease severity, residual confounding cannot be eliminated. The calculated E-values exceed the observed HRs, suggesting that minor unmeasured confounders are unlikely to fully explain the observed associations and indicating that these findings may reflect a meaningful relationship rather than establish direct causality.

## Conclusion

In this extensive retrospective cohort study, mitral valve surgery and TEER demonstrated differing outcome profiles. Short-term mortality appeared similar between the two strategies, while surgery showed a trend toward better survival beyond 2 months. Surgery, however, was associated with a higher early incidence of dyspnea, atrial fibrillation, and worsening heart failure, whereas TEER was linked to more favorable early symptomatic outcomes. No significant differences were noted in MACE, stroke, AMI, or ED visits. Overall, these findings offer real-world insights that may help guide more individualized treatment decisions for patients with non-rheumatic MR.

## Data Availability

The original contributions presented in the study are included in the article/Supplementary material, further inquiries can be directed to the corresponding author.
