# Supplementary material for: A real-world comparison of transcatheter edge-to-edge repair versus surgical mitral valve intervention in patients with mitral regurgitation: a TriNetX study
Source: Front Med (Lausanne). 2026 Feb 9;13:1729871. doi: 10.3389/fmed.2026.1729871 (PMC12926431; doi:10.3389/fmed.2026.1729871)
Supplement: Supplementary file 1 [file Supplementary_file_1.docx]

**Table S1.** Demographic, diagnostic, procedural, medication, visit, and laboratory codes utilized in the definition of the cohorts.

| **Category** | **Code** | **Description** |
| --- | --- | --- |
| **Surgery** | | |
| procedure | UMLS:CPT:33427 | Valvuloplasty, mitral valve, with cardiopulmonary bypass; radical reconstruction, with or without ring |
| procedure | UMLS:CPT:33425 | Valvuloplasty, mitral valve, with cardiopulmonary bypass |
| procedure | UMLS:CPT:33426 | Valvuloplasty, mitral valve, with cardiopulmonary bypass; with prosthetic ring |
| procedure | UMLS:CPT:33430 | Replacement, mitral valve, with cardiopulmonary bypass |
| procedure | UMLS:CPT:33463 | Valvuloplasty, tricuspid valve; without ring insertion |
| procedure | UMLS:CPT:33418 | Transcatheter mitral valve repair, percutaneous approach, including transseptal puncture when performed; initial prosthesis |
| procedure | UMLS:CPT:33419 | Transcatheter mitral valve repair, percutaneous approach, including transseptal puncture when performed; additional prosthesis(es) during same session (List separately in addition to code for primary procedure) |
| **TEER** | | |
| procedure | UMLS:CPT:33418 | Transcatheter mitral valve repair, percutaneous approach, including transseptal puncture when performed; initial prosthesis |
| procedure | UMLS:ICD10PCS:02UG3JZ | Supplement Mitral Valve with Synthetic Substitute, Percutaneous Approach |
| procedure | UMLS:CPT:33419 | Transcatheter mitral valve repair, percutaneous approach, including transseptal puncture when performed; additional prosthesis(es) during same session (List separately in addition to code for primary procedure) |
| **Nonrheumatic mitral (valve) prolapse and insufficiency** | | |
| diagnosis | UMLS:ICD10CM:I34.1 | Nonrheumatic mitral (valve) prolapse |
| diagnosis | UMLS:ICD10CM:I34.0 | Nonrheumatic mitral (valve) insufficiency |

**Table S2.** Definitions of covariates coding used in this study.

| **Code** | **Description** |
| --- | --- |
| AI | Age at Index |
| 2106-3 | White |
| UNK | Unknown Race |
| F | Female |
| 2054-5 | Black or African American |
| M | Male |
| 2131-1 | Other Race |
| 2028-9 | Asian |
| F17 | Nicotine dependence |
| F10 | Alcohol related disorders |
| E66 | Overweight and obesity |
| I10 | Essential (primary) hypertension |
| E78 | Disorders of lipoprotein metabolism and other lipidemias |
| E11 | Type 2 diabetes mellitus |
| N18 | Chronic kidney disease (CKD) |
| I60-I69 | Cerebrovascular diseases |
| J40-J4A | Chronic lower respiratory diseases |
| I20-I25 | Ischemic heart diseases |
| I10-I1A | Hypertensive diseases |
| C00-D49 | Neoplasms |
| 1649480 | ivabradine |
| CV700 | DIURETICS |
| CV800 | ACE INHIBITORS |
| CV805 | ANGIOTENSIN II INHIBITOR |
| 298869 | eplerenone |
| 2475830 | vericiguat |
| C01AA | Digitalis glycosides |
| C10AA | HMG CoA reductase inhibitors |
| 341248 | ezetimibe |
| 1665684 | evolocumab |
| 1659152 | alirocumab |
| 9037 | Hemoglobin A1c/Hemoglobin.total in Blood |

**Table S3.** Definitions of outcomes coding used in this study.

| **Code** | **Description** |
| --- | --- |
| **All-cause mortality** | |
| Deceased | Deceased |
| R99 | Ill-defined and unknown cause of mortality |
| **All-cause ED visit** | |
| Visit: Emergency | Visit: Emergency |
| **Dyspnea** | |
| R06.0 | Dyspnea |
| **Mitral stenosis** | |
| I34.2 | Nonrheumatic mitral (valve) stenosis |
| **Atrial fibrillation** | |
| I48 | Atrial fibrillation and flutter |
| **MACEs** | |
| I21 | Acute myocardial infarction |
| I63 | Cerebral infarction |
| I46 | Cardiac arrest |
| **Stroke** | |
| I63 | Cerebral infarction |
| **AMI** | |
| I21 | Acute myocardial infarction |
| **Cardiac arrest** | |
| I63 | Cerebral infarction |
| **Worsening heart failure** | |
| CV701 | THIAZIDES/RELATED DIURETICS (Route: Injectable Product) |
| CV702 | LOOP DIURETICS (Route: Injectable Product) |
| J81 | Pulmonary edema |

**Table S4.** Hazard ratios of outcomes between the surgery and the TEER groups at 1-day and 1-month follow-up after the index date.

| Outcome | Surgery group (n = 2,029) | | TEER group (n = 2,029) | | HR (95% CI) | *P* value | E-value (95% LCL) |
| --- | --- | --- | --- | --- | --- | --- | --- |
|  | Events (n) | Incidence rate  per 100 PYs | Events (n) | Incidence rate  per 100 PYs |  |  |  |
| Primary outcome |  |  |  |  |  |  |  |
| All-cause mortality | 66 | 39.6 | 251 | 150.6 | 1.30 (0.90,1.87) | 0.160 | 1.9 (1.0) |
| Secondary outcomes |  |  |  |  |  |  |  |
| All-cause ED visit | 37 | 22.2 | 19 | 11.4 | 1.70 (0.98,2.95) | 0.058 | 2.8 (1.0) |
| Dyspnea | 95 | 57.0 | 39 | 23.4 | 2.67 (1.84,3.87) | <0.001 | 4.8 (3.1) |
| Mitral stenosis | 13 | 7.8 | 19 | 11.4 | 0.73 (0.36,1.47) | 0.375 | 2.1 (1.0) |
| Atrial fibrillation | 97 | 58.2 | 90 | 54.0 | 1.95 (1.46,2.59) | <0.001 | 3.3 (2.3) |
| MACEs | 65 | 39.0 | 37 | 22.2 | 1.74 (1.16,2.61) | 0.006 | 2.9 (1.6) |
| Stroke | 35 | 21.0 | 17 | 10.2 | 2.02 (1.13,3.60) | 0.015 | 3.5 (1.5) |
| AMI | 40 | 24.0 | 18 | 10.8 | 2.20 (1.26,3.84) | 0.004 | 3.8 (1.8) |
| Cardiac arrest | - | - | - | - | - | - | - |
| Worsening heart failure | 708 | 424.8 | 106 | 63.6 | 8.09 (6.59,9.92) | <0.001 | 15.7 (12.7) |

**Table S5.** Hazard ratios of outcomes between the surgery and the TEER groups at 1-month and 1-year follow-up after the index date.

| Outcome | Surgery group (n = 2,029) | | TEER group (n = 2,029) | | HR (95% CI) | *P* value | E-value (95% LCL) |
| --- | --- | --- | --- | --- | --- | --- | --- |
|  | Events (n) | Incidence rate  per 100 PYs | Events (n) | Incidence rate  per 100 PYs |  |  |  |
| Primary outcome |  |  |  |  |  |  |  |
| All-cause mortality | 95 | 7.0 | 178 | 13.1 | 0.56 (0.43,0.71) | <0.001 | 3.0 (2.2) |
| Secondary outcomes |  |  |  |  |  |  |  |
| All-cause ED visit | 78 | 5.7 | 80 | 5.9 | 0.91 (0.66,1.24) | 0.533 | 1.4 (1.0) |
| Dyspnea | 74 | 5.4 | 91 | 6.7 | 0.99 (0.73,1.35) | 0.964 | 1.1 (1.0) |
| Mitral stenosis | - | - | - | - | - | - | - |
| Atrial fibrillation | 34 | 2.5 | 78 | 5.7 | 0.86 (0.57,1.28) | 0.453 | 1.6 (1.0) |
| MACEs | 52 | 3.8 | 54 | 4.0 | 1.00 (0.69,1.47) | 0.984 | 1.0 (1.0) |
| Stroke | 26 | 1.9 | 24 | 1.8 | 1.10 (0.63,1.92) | 0.729 | 1.4 (1.0) |
| AMI | 31 | 2.3 | 34 | 2.5 | 0.95 (0.58,1.54) | 0.828 | 1.3 (1.0) |
| Cardiac arrest | 11 | 0.8 | 11 | 0.8 | 1.04 (0.45,2.41) | 0.921 | 1.2 (1.0) |
| Worsening heart failure | 20 | 1.5 | 55 | 4.0 | 0.69 (0.42,1.16) | 0.159 | 2.3 (1.0) |

**Table S6.** Sensitivity analysis of outcomes restricted to procedures performed in 2018–2025.

| Outcome | Surgery group (n = 2,029) | | TEER group (n = 2,029) | | HR (95% CI) | *P* value | E-value (95% LCL) |
| --- | --- | --- | --- | --- | --- | --- | --- |
|  | Events (n) | Incidence rate  per 100 PYs | Events (n) | Incidence rate  per 100 PYs |  |  |  |
| Primary outcome |  |  |  |  |  |  |  |
| All-cause mortality | 108 | 5.5 | 187 | 9.5 | 0.58 (0.45,0.73) | <0.001 | 2.8 (2.1) |
| Secondary outcomes |  |  |  |  |  |  |  |
| All-cause ED visit | 117 | 6.0 | 84 | 4.3 | 1.25 (0.94,1.65) | 0.119 | 1.8 (1.0) |
| Dyspnea | 162 | 8.2 | 111 | 6.7 | 1.67 (1.32,2.13) | <0.001 | 2.7 (2.0) |
| Mitral stenosis | 19 | 1.0 | 43 | 2.2 | 0.46 (0.27,0.79) | 0.004 | 3.8 (1.9) |
| Atrial fibrillation | 111 | 5.6 | 148 | 7.5 | 1.36 (1.06,1.74) | 0.015 | 2.1 (1.3) |
| MACEs | 115 | 5.9 | 83 | 4.2 | 1.38 (1.04,1.83) | 0.026 | 2.1 (1.2) |
| Stroke | 61 | 3.1 | 39 | 2.0 | 1.55 (1.03,2.31) | 0.032 | 2.5 (1.2) |
| AMI | 65 | 3.3 | 47 | 2.4 | 1.36 (0.94,1.98) | 0.106 | 2.1 (1.0) |
| Cardiac arrest | 17 | 0.9 | 18 | 0.9 | 0.94 (0.49,1.83) | 0.862 | 1.3 (1.0) |
| Worsening heart failure | 701 | 35.7 | 145 | 7.4 | 6.52 (5.45,7.81) | <0.001 | 12.5 (10.4) |
